# Antidepressants for depressive disorder in children and adolescents: a database of randomised controlled trials

**DOI:** 10.1186/s12888-018-1749-0

**Published:** 2018-05-31

**Authors:** Yuqing Zhang, Xinyu Zhou, Juncai Pu, Hanping Zhang, Lining Yang, Lanxiang Liu, Chanjuan Zhou, Shuai Yuan, Xiaofeng Jiang, Peng Xie

**Affiliations:** 1 Department of Neurology, Yongchuan Hospital of Chongqing Medical University, Chongqing, China; 2 Chongqing Key Laboratory for Cerebrovascular Disease Research, Yongchuan Hospital of Chongqing Medical University, Chongqing, China; 3 Department of Psychiatry, The First Affiliated Hospital of Chongqing Medical University, Chongqing, China; 4 Department of Neurology, The First Affiliated Hospital of Chongqing Medical University, 1 Youyi Road, Yuzhong District, Chongqing, 400016 China

**Keywords:** Depressive disorder, Children, Adolescents, Antidepressants, Randomised controlled trials, Database, Meta-analysis, Systematic review

## Abstract

**Background:**

In recent years, whether, when and how to use antidepressants to treat depressive disorder in children and adolescents has been hotly debated. Relevant evidence on this topic has increased rapidly. In this paper, we present the construction and content of a database of randomised controlled trials of antidepressants to treat depressive disorder in children and adolescents. This database can be freely accessed via our website and will be regularly updated.

**Description:**

Major bibliographic databases (PubMed, the Cochrane Library, Web of Science, Embase, CINAHL, PsycINFO and LiLACS), international trial registers and regulatory agencies’ websites were systematically searched for published and unpublished studies up to April 30, 2017. We included randomised controlled trials in which the efficacy or tolerability of any oral antidepressant was compared with that of a control group or any other treatment. In total, 7377 citations from bibliographical databases and 3289 from international trial registers and regulatory agencies’ websites were identified. Of these, 53 trials were eligible for inclusion in the final database. Selected data were extracted from each study, including characteristics of the participants (the study population, setting, diagnostic criteria, type of depression, age, sex, and comorbidity), characteristics of the treatment conditions (the treatment conditions, general information, and detail of pharmacotherapy and psychotherapy) and study characteristics (the sponsor, country, number of sites, blinding method, sample size, treatment duration, depression scales, other scales, and primary outcome measure used, and side-effect monitoring method). Moreover, the risk of bias for each trial were assessed.

**Conclusion:**

This database provides information on nearly all randomised controlled trials of antidepressants in children and adolescents. By using this database, researchers can improve research efficiency, avoid inadvertent errors and easily focus on the targeted subgroups in which they are interested. For authors of subsequent reviews, they could only use this database to insure that they have completed a comprehensive review, rather than relied solely on the data from this database. We expect this database could help to promote research on evidence-based practice in the treatment of depressive disorder in children and adolescents. The database could be freely accessed in our website: http://xiepengteam.cn/research/evidence-based-medicine.

## Background

Depressive disorder is common in children and adolescents. Untreated episodes of depressive disorder in these groups frequently result in serious impairments in terms of personal and social functioning [1, 2]. Although some psychological treatments are demonstrated efficacious [3–5], many young people cannot access this kind of treatment soon enough [6]. For this reason, antidepressants are widely used in children and adolescents, with the prescription of these drugs continuing to increase in recent years [7].

In the past 20 years, the number of trials and review articles investigating the efficacy and tolerability of antidepressants in the treatment of depressive disorder in children and adolescents has increased rapidly. Most of the meta-analyses of this work have focused on one specific subgroup of studies, such as a specific class of antidepressants [8–11], a specific population [12–14] or a specific mode of therapy [15–17]. A recent network meta-analysis in which the authors participated found a surprising result that the risk–benefit profile of 14 included antidepressants in the acute treatment of depression did not seem to offer a clear advantage of using these drugs for children and adolescents [18]. However, this result was limited by the low quality of evidence for most of the comparisons and influenced by potential moderators (e.g., the implementation deficits among studies [19]). Thus, the questions of whether antidepressants are effective and safe for children and adolescents with depressive disorder and which is the most suitable drug for different subgroups among these populations remain uncertain. It is vital for us to constantly update the evidence and consolidate our knowledge in this field to better support clinical decisions.

Our group is engaged in research on evidence-based medicine for depression in children and adolescents. In the past 5 years, we have built a database of all randomised controlled trials (RCTs) of antidepressants in children and adolescents with depressive disorder. Using subgroups of studies in this database, we have published five meta-analyses focusing on different topic [10–12, 14, 18]. In this paper, we present the method and process of establishing the database and briefly introduce the characteristics of the included studies. Other researchers can easily access the dataset and use it for further analysis. The database can be freely accessed in our website: http://xiepengteam.cn/research/evidence-based-medicine.

## Construction and content

### Identification and selection of studies

We conducted a comprehensive search of seven electronic databases (PubMed, the Cochrane Library, Web of Science, Embase, CINAHL, PsycINFO and LiLACS) for RCTs published from the date of each database’s inception to April 31, 2017. The following words and Medical Subject Headings (MeSH) were searched with a filter for clinical trials: (depress* or dysthymi* or mood disorder* or affective disorder*) AND (adolesc* or child* or boy* or girl* or juvenil* or minors or paediatri* or pediatri* or pubescen* or school* or student* or teen* or young or youth*) AND (antidepressant* or selective serotonin reuptake inhibitor* or SSRI or SSRIs or citalopram or fluoxetine or paroxetine or sertraline or escitalopram or fluvoxamine or serotonin norepinephrine reuptake inhibitor* or SNRI or SNRIs or venlafaxine or duloxetine or milnacipran or reboxetine or bupropion or noradrenergic and specific serotonergic antidepressant* or NaSSA or NaSSAs or mirtazapine or nefazodone or trazodone or TCA or TCAs or tricyclic or amersergide or amineptine or amitriptyline or amoxapine or butriptyline or chlorpoxiten or clomipramine or clorimipramine or demexiptiline or desipramine or dibenzepin or dothiepin or doxepin or imipramine or lofepramine or melitracen or metapramine or nortriptyline or noxiptiline or opipramol or protriptyline or quinupramine or tianeptine or trimipramine). No restrictions were set on language. In addition, international trial registers and a regulatory agency’s website (US Food and Drug Administration (FDA)) were searched for published and unpublished studies.

We began the project and conducted the original searches for several subgroups of studies in December 2013. We then conducted another search with more comprehensive search terms (listed above) for all studies on May 31, 2015. We updated the search on April 30, 2017. Table 1 presents the number of citations identified from each bibliographic database and trial register. In total, 7377 citations were identified from the bibliographic databases, and 3289 were identified from the international trial registers and the FDA website. Further, relevant principal manufacturers (e.g. GlaxoSmithKline, Lilly, Organon, Forest Pharmaceuticals, Bristol-Myers Squibb) were contacted, and some relevant journals and conference proceedings were manually searched. Additional relevant studies were obtained by scanning the reference lists of relevant systematic reviews, meta-analyses and eligible trials [20].

**Table 1:** Number of abstracts identified at each search

Databases and Trial registers:	Titles and abstracts	
	First search Published before May 2015	Updated May 2015–April 2017
Databases		
PubMed	364	341
Cochrane	1556	209
Web of Science	1743	406
Embase	638	284
CINAHL	172	31
PsychInfo	1277	288
LILACS	44	24
Total (databases)	5794	1583
Trial registers		
Australia (ANZCTR)	108	15
China (ChiCTR)	12	2
USA (ClinicalTrials.gov)	214	15
Japan (UMIN-CTR)	56	10
Netherlands (Trial Register)	14	3
UN (ISRCTN)	110	9
World Health Organization (ICTRP)	1003	154
USA Food and Drug Administration (FDA)	992	572
Total (trial registers)	2509	780

### Inclusion of studies

For the database, we selected the studies in which (1) children and adolescents (aged 6–18 years at initial trial enrolment) were included, (2) a primary diagnosis of current depressive disorder was confirmed by standardised diagnostic interviews based on international classifications (e.g. the Diagnostic and Statistical Manual of Mental Disorders, and the International Classification of Diseases [21–25]), (3) the efficacy or tolerability of an oral antidepressant or combined therapy (pharmacotherapy plus psychotherapy) was compared with that of a control condition or any other treatment, and (4) a completely randomised design was adopted.

Antidepressants can be divided into several classes, for example, tricyclic antidepressants (TCAs), selective serotonin reuptake inhibitors (SSRIs), serotonin–norepinephrine reuptake inhibitors (SNRIs), and noradrenergic and specific serotonergic antidepressants (NaSSAs). In this database, RCTs comparing any antidepressant with an active comparator or placebo for the treatment of depressive disorder in children and adolescents were included, regardless of the class of drug, dose range and treatment duration. Trials including participants with any psychiatric comorbidity or physical disease were also selected for inclusion.

### Screening process

**Fig. 1:**
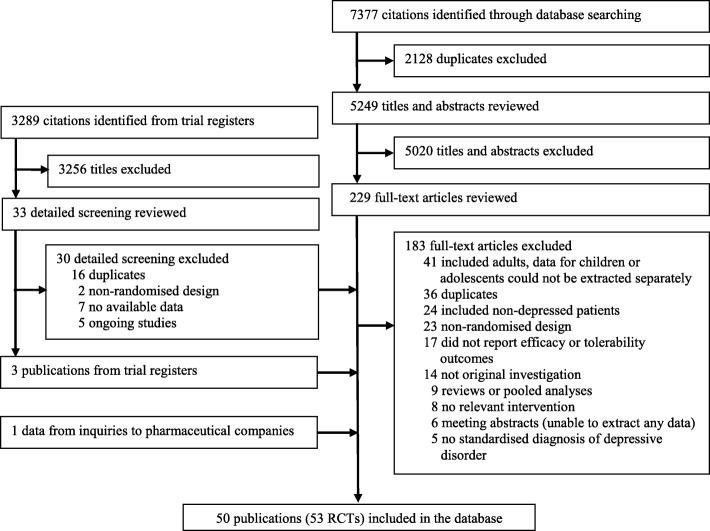
Process of literature search and study selection. RCT = randomised controlled trial

We identified 7377 potentially relevant studies from the bibliographic databases. After removing 2128 duplicate records, 5249 titles and abstracts were reviewed by two independent reviewers. Of these, 5020 studies were excluded because they did not meet the inclusion criterion. The full text of the remaining 229 articles was reviewed. From these, 46 articles were deemed eligible, and 183 were excluded from the final database. In terms of international trial registers and the FDA website, 3289 citations were initially identified. Two independent reviewers independently scanned the titles, and 3256 studies were excluded. Next, 33 records were reviewed in detail. Finally, three eligible publications from trial registers and the FDA website were included. Additionally, we obtained further studies from inquiries to pharmaceutical companies. In total, 50 publications (reporting the results of 53 RCTs) were included in the final database. All disagreements between reviewers in the screening process were resolved through discussion with a senior reviewer (PX or XZ) in the team. Figure 1 presents a flowchart illustrating the screening process in detail.

### Data extraction

At least two reviewers independently extracted the key parameters from each included study using a standardised data abstraction form. Any disagreements were resolved through discussion with a senior reviewer (PX or XZ) in the team. Although different meta-analyses focus on different characteristics, we summarised and extracted the following important items for each trial:

#### Characteristics of participants

- Population: In this column, the targeted population, such as children (aged 6–12 years) or adolescents (aged 13–18 years) in general, adolescents with treatment-resistant depression or adolescents with substance use disorder, is described in each trial.
- Setting: This column indicates whether outpatients or inpatients were recruited for the study.
- Diagnostic criteria: Here, we report the diagnostic criteria used for the diagnosis of depressive disorder in children and adolescents.
- Type of depression: In this column, we describe the types of depression included (major depressive disorder, dysthymic disorder, depressive disorder not otherwise specified, minor depression, etc.) and the proportion of patients with each type in the study.
- Age: Participants’ age is reported here, with the range and mean presented in two columns.
- Sex: Here, we report the proportion of participants who are female.
- Comorbidity: This column describes the types of psychiatric comorbidity of participants, and if available, the proportion of participants in each comorbidity is also reported.

#### Characteristics of treatment conditions

- Conditions: Here, we briefly describe all the conditions examined in each trial. This column provides an overall impression of the group assignment in each trial.
- General: Here, for each trial, we describe the general information about every condition included. In the ‘Type’ column, we describe the general types of conditions, such as pharmacotherapy, psychotherapy, combined therapy or control condition. In the ‘N baseline’ column, we report the number of participants randomly assigned to each condition.
- Pharmacotherapy: In these columns, we describe the name, class (such as SSRI, SNRI, TCA or NaSSA) and dose range of the antidepressants used in the condition of pharmacotherapy or combined therapy.
- Psychotherapy: In these columns, we describe the characteristics of the psychotherapies used in the condition of psychotherapy or combined therapy. The ‘Type’ column refers to the type of psychotherapy (cognitive behaviour therapy, interpersonal therapy, family therapy, etc.) [3]. The ‘Format’ column indicates the format used in the psychotherapy (e.g. individual therapy, group therapy, bibliotherapy or Internet-assisted therapy). In the ‘Number of sessions’ column, we report the number of psychotherapy sessions.

#### Study characteristics

- Sponsor: Here, we indicate the sponsor of the trial, which may include pharmaceutical industries and non-profit organisations.
- Country: In this column, we report the country where the trial was conducted.
- Number of sites: This column report the number of sites in each trial.
- Blinding: The blinding method used in each trial is indicated in this column, including ‘Double-blind’, ‘Single-blind’ and ‘Non-blind’.
- Sample size: This column refers to the total number of randomly assigned participants in each trial.
- Treatment duration: In this column, the total treatment duration is reported. If a trial conducted extension treatment, we also indicate the acute treatment duration.
- Depression scales: In this column, we report the main depression measurement scales that the study used to measure depressive symptom severity.
- Other scales: Here, we report other important scales used in each trial.
- Primary outcome measure: This column indicates what was used as the primary outcome measure in each trial.
- Side-effect monitoring: In this column, the method of side-effect monitoring in each trial is briefly descript as ‘spontaneous’, ‘unstructured’, ‘structured’, and ‘measured with ...’.

We did not include the original quantitative values of the measures or the effect sizes in this database, because different meta-analyses may require different statistical approaches and the corresponding datasets. Our main purpose was not to extract all data from each study, but rather to provide a comprehensive overview and tell other researchers what they can do. Thus, we only reported the kind of measures used in each trial.

#### Risk of bias assessment

Two investigators independently assessed the risk of bias of included trials using the Cochrane risk of bias tool [26]. The risk of bias was rated as ‘L’(low risk), ‘U’(unclear risk) or ‘H’(high risk) within the following items: sequence generation, allocation concealment, blinding of participants and personnel, blinding of outcome assessors, incomplete outcome data, selective outcome reporting and other sources of bias. As it is usually not possible to perform blindness for participants and personnel in psychotherapy trials, this term was rated as high risk in all these trials. It is noted that the risk of bias tool could not cover each aspect of bias, which may include the information about who the investigators were and their specific expertise, how selected, how trained, how monitored over the course of the study for maintaining quality in recruitment, retention, etc. These are also an important area of bias but usually hard to assess.

## Utility

This database includes nearly all RCTs of depressive disorder in children and adolescents that compare the efficacy or tolerability of an intervention involving any antidepressant with that of a control group or any other treatment. Using this database, other researchers can not only save a large amount of time through the searching, screening and checking publications from various resources that we have done, but also avoid inadvertent omissions and overlaps with existing studies. In addition, this database will make it convenient for other reviewers to re-analyse the results from previous meta-analyses independently; this will increase the transparency and reliability of the methods used in research by different review teams.

In addition, this database describes the characteristics of studies, participants and interventions in detail. This will allow other researchers to quickly get a comprehensive overview of the existing evidence regarding pharmacotherapy for depressive disorder in children and adolescents. It will also help scholars to focus on the specific subgroups they interested, such as studies in which patients with similar characteristics, interventions with same types or classes, and outcomes with same measurements. By pooling these similar studies, more targeted systematic reviews and meta-analyses, with less mistakes and incorrect estimations, will be produced. This will finally promote the optimisation of clinical decision making.

Furthermore, the risk of bias of included trials has been assessed within Cochrane risk of bias tool. However, it is important to note that the methodology of RCTs progressed over time, resulting that the quality of trial in this database varied dramatically from early TCA trials to more recent SSRI trials. Thus, researchers using this database should not simply group poor quality studies and make poor quality conclusions. Reasonable subgroup analyses, meta-regression or sensitive analyses according to risk of bias and publication year are suggested. In addition, the interpretation of such results should be in caution.

Considering that this field has attracted a great deal of attention and that relevant studies have increased rapidly in recent years, we will continue to update this database by keep searching the seven major literature databases, the major international trial registers and FDA reports. In addition, we will continue to retrieve reference lists of the relevant studies and contact manufacturers for industry data. The update time will be between May and July in each year. And the update results will be reported in our website. Moreover, we will continue to search and check potentially eligible studies that were omitted in previous searches.

## Discussion

In 2004, the US FDA cautioned that the use of antidepressants in children and adolescents may be associated with increased risk for suicidality [27]. In addition, a recent network meta-analysis found that most antidepressants may ineffective for children and adolescents with depressive disorder [18]. Therefore, whether antidepressants are effective and safe for the treatment of depression in children and adolescents has caused great concern in the recent years. However, from limited number and poor quality of the current evidence, we could not draw a reliable conclusion. Thus, a constantly updated database, which collected and will continue to collect all relevant RCTs in this field, will be helpful for us to find this answer in the future.

This paper presented the construction method and process of a free online database of RCTs of antidepressants for treating depressive disorder in children and adolescents. The content and utility of this database were also described. By using this database, researchers could quickly find the evidence regarding their interested antidepressants. It is noted that the purpose of this paper was not to analyse the studies included in this database, but rather to provide a description of what is currently the most comprehensive resource of RCTs in this field and to illustrate what this resource can do.

Similar databases, such as the Cuijpers et al. database of psychological treatment for adults with depression [28] and the Christensen et al. database of psychosocial interventions for suicidal ideation, plans and attempts [29], have been highly cited and have effectively stimulated an increase in the quantity and quality of relevant reviews. Therefore, we have reason to believe that the database described in the present paper will also be useful for future research.

The database does have some limitations. First, there is also a sizable study of antidepressants and psychotherapy for anxiety disorder and obsessive-compulsive disorder (OCD). However, we only included the literature of antidepressants and part of psychotherapies for depressive disorder. Therefore, this database is only part of the antidepressant and psychotherapy picture for internalizing conditions. Second, this database only collected the original RCT reports. Whereas some RCTs can derive numerous papers that tell more comprehensive stories of these studies, the original RCT reports are only a limited section of these depression literatures. Third, although we attempted to retrieve all available published and unpublished studies that were eligible for this database, we cannot rule out the possibility that some studies are still missing. Fourth, it is possible that some characteristics of interest to other researchers are not included in the current version of the database. Thus, we will continuously review and expand the content of this database. We sincerely welcome suggestions from other reviewers. Fifth, although we have tried to avoid errors, we cannot be certain that all of the data are entirely complete and accurate. Thus, if anyone found a mistake or an omitted study that meets our inclusion criteria, please contact us via email: xiepeng973@126.com. Finally, it is possible that some caveats indicated in this paper will not be applicable in the future, and some future problems will be identified in the updated literature. Therefore, we will continue to update the caveats in our website: http://xiepengteam.cn/research/evidence-based-medicine.

## Conclusions

The present database collects nearly all randomised controlled trials of antidepressants for treating depressive disorder in children and adolescents. By using the comprehensive and well maintained database, researchers can improve research efficiency, avoid inadvertent errors and easily focus on their interested subgroups. This database will help to promote the performance of high-quality evidence-based studies in this field, as well as the optimisation of clinical decisions on antidepressants for children and adolescents with depressive disorder.

## Abbreviations

NaSSAs: Noradrenergic and specific serotonergic antidepressants
RCT: Randomised controlled trial
SNRIs: Serotonin–norepinephrine reuptake inhibitors
SSRIs: Selective serotonin reuptake inhibitors
TCAs: Tricyclic antidepressants
